# Bio-functionalized magnetic nanoparticles for the immunoassay of fetal fibronectin: a feasibility study for the prediction of preterm birth

**DOI:** 10.1038/srep42461

**Published:** 2017-02-15

**Authors:** Chian-Huey Wong, Chie-Pein Chen, Chia-Chen Chang, Chen-Yu Chen

**Affiliations:** 1 Department of Obstetrics and Gynecology, Mackay Memorial Hospital, Taipei, Taiwan; 2 Department of Medicine, Mackay Medical College, New Taipei City, Taiwan; 3 Institute of Biomedical Engineering, National Taiwan University, Taipei, Taiwan

## Abstract

Preterm birth is an important cause of perinatal morbidity and mortality. Various biomarkers in cervicovaginal secretions related to preterm birth have been investigated, of which foetal fibronectin (fFN) shows the greatest potential because of its high negative predictive value. The immunomagnetic reduction (IMR) assay has emerged as a novel quantitative method to detect biomarkers. In this prospective case-control study, we analysed 33 samples of cervicovaginal secretions from pregnant women between 22 and 34 weeks of gestation at high risk of preterm birth. Seventeen samples were from women with term deliveries and 16 from those with preterm deliveries. The fFN concentration in each sample was measured using both an IMR assay and enzyme-linked immunosorbent assay (ELISA). The low detection limits of the IMR assay and ELISA were 0.0001 ng/mL and 0.789 ng/mL, respectively. The sensitivity and specificity of the IMR assay were 0.833 and 0.944, respectively, compared to 0.583 and 0.611 by ELISA. Our results suggest that measuring the concentration of fFN with the IMR assay is a good alternative method to accurately predict the risk of preterm birth.

Preterm birth is defined as delivery at less than 37 weeks of gestation^1^. It is the leading cause of perinatal morbidity and mortality worldwide, occurring in 5% to 13% of pregnancies in developed countries and accounting for 70% of neonatal deaths and half of all neonatal neurological complications^2^,^3^,^4^,^5^. The incidence of preterm birth continues to increase despite substantial efforts focused on prevention including advances in technology and increasingly well-trained healthcare professionals^6^. Therefore, further methods to predict preterm birth are urgently needed.

In current obstetric practice, preterm labour is mainly diagnosed by the presence of regular painful uterine contractions accompanied by cervical dilatation or effacement before 37 weeks of gestation. However, the sensitivity and specificity of these signs are low in most cases, and this can impact clinical decision making with regards to identifying patients at risk of preterm birth. Numerous studies have explored the influence of various biomarkers on the diagnosis of preterm birth, and foetal fibronectin (fFN) has been shown to be particularly useful in identifying those at high risk^7^,^8^. fFN is an extracellular glycoprotein which is initially contained in the choriodecidual space. It is released into cervicovaginal secretions when disruption of the choriodecidual interface occurs secondary to shear forces induced by uterine contractions or degradation caused by inflammatory processes. Since fFN is almost undetectable between the second and early third trimesters of pregnancy, the use of fFN in excluding preterm labour is enhanced by its high negative predictive value^9^,^10^. It has been reported that patients with a fFN concentration ≥50 ng/mL are at a higher risk of preterm birth^11^. The US Food and Drug Administration (FDA) has approved a fFN enzyme-linked immunosorbent assay (ELISA) and immunochromatographic assay (TLi system, Adeza Biomedical Corporation, Sunnyvale, CA, USA) for use in the prediction of preterm birth. However, there are some limitations of these two conventional methods. The ELISA method is time-consuming, and potential cross-reactions between bound antibodies can result in inaccurate colour signal intensity measurements. In addition, an immunochromatographic assay is not a quantitative method, and it cannot predict the severity of disease. Therefore, a rapid, label-free, and quantitative assay is needed.

Recently, many innovate methods have been developed to measure fFN concentrations, such as aptamer-based immunoassays, surface plasmon resonance biosensors, and immunomagnetic reduction (IMR) assays^12^,^13^,^14^. The IMR assay has emerged as a novel quantitative method to detect biomarkers, and it has been shown to enhance sensitivity and specificity of biomarker detection^15^,^16^,^17^. It measures the concentration of fFN by comparing changes in magnetic responses between free and conjugated magnetic nanoparticles. In the previous study we presented the preparation of antibody functionalized magnetic nanoparticles, and demonstrated the bio-activity of these nanoparticles in associating with fFN^14^. Furthermore, the preliminary results showed the possibility to assay fFN in phosphate buffered solution (PBS) via IMR technology. Although these findings reveal the possibility of precise fFN assay using IMR technology, the detailed relationship between the IMR signal and fFN concentration is lack. Besides, the understanding of assaying fFN in human samples using IMR is poor. More explorations in the feasibility of assaying fFN, as well as in correlating the fFN concentration in human samples to predict the risk of preterm birth, are absolutely needed. In the current study, we investigated whether the IMR assay can be used to detect fFN concentrations in cervicovaginal secretions collected from pregnant women at high risk of preterm birth. In addition, we compared the IMR assay and ELISA in their ability to detect fFN.

## Methods

### Sample collection

We conducted this prospective case-control study at Mackay Memorial Hospital, Taipei, Taiwan from July 2014 to March 2015. We enrolled pregnant women with a gestational age between 22 and 34 weeks who presented to our emergency room with symptom of preterm uterine contractions, defined as regular and frequent uterine tightening or cramping. Routine sterile speculum examinations were performed to confirm cervical dilatation. Cervicovaginal secretions were obtained before other procedures such as vaginal probe ultrasound and endocervical cultures to prevent sample contamination. The standard protocol for sample collection was as follows: one sterile cotton swab was placed at the posterior fornix of the vagina for 10 seconds with gentle rotation to ensure adequate collection. The cotton swab was then placed in a tube containing 3 ml of PBS buffer (pH 7.4) and then mixed for 10 seconds before being sent for IMR and ELISA analyses. The patients with cervical dilatation of >4 cm and/or with evidence of membrane rupture were excluded from the study. The enrolled patients were followed throughout their pregnancy, and their gestational age at delivery was recorded. The patients with a gestational age of ≥37 weeks were categorized into the negative group, and those with a gestational age <37 weeks were categorized into the positive group. Clinically, women in the negative group had term deliveries, while those in the positive group had preterm deliveries. This study was approved by the Mackay Memorial Hospital Institutional Review Board (IRB #09MMHIS056), and the methods were carried out in accordance with the approved guidelines. Informed consents were obtained from all of the enrolled women for the collection and examination of clinical samples.

### Preparation of magnetic reagents

The magnetic nanoparticles (MagQu, New Taipei City, Taiwan) were synthesized via chemical co-precipitation^18^. The processes of synthesizing magnetic nanoparticles are described as follows. A stoichiometric ratio of 1:2 of ferrous sulphate heptahydrate (FeSO_4_·7H_2_O) and ferric chloride hexahydrate (FeCl_3_·6H_2_O) magnetic fluid was mixed with the same proportion of aqueous dextran, which was used as a surfactant for magnetic Fe_3_O_4_ nanoparticles. The mixture was heated gently up to 80–100 °C. After that, during rigorous stirring, the mixture was titrated to have a pH of around 10–11 at room temperature. It can be observed that the solution became black due to the formation of Fe_3_O_4_ nanoparticles. The black mixture was then heated at 60–80 °C in a water bath to coat the Fe_3_O_4_ nanoparticles with dextran. The excess unbound dextran was separated by gel filtration chromatography. The purified water-based magnetic fluid containing dextran-coated Fe_3_O_4_ nanoparticles collected in the void volume had a concentration of about 8 mg-Fe/mL. The detailed examination for the crystalline of the synthesized magnetic nanoparticles by using θ–2θ powder x-ray diffraction has been studied, and the x-ray pattern reveals that only Fe_3_O_4_ phase was observed for the magnetic nanoparticles^18^. The mean (standard deviation) hydrodynamic diameter of dextran-coated Fe_3_O_4_ was measured as 40.22 nm (9.23 nm) by using dynamic laser scattering (DLS; Nanotrac 150, Microtrac, PA, USA). A previous similar study for demonstrating the coating of dextran/antibody on the magnetic nanoparticle by transmission electron microscopy (TEM) revealed that the core-shell structure is clear in the TEM image^19^.

To bio-functionalize the magnetic nanoparticles with antibodies against fFN, denoted as anti-fFN (ab18265; Abcam, Cambridge, UK), aldehyde groups (–CHO) were initially formed on dextran via an oxidation reaction^20^. These aldehyde groups then reacted with the anti-fFN to form –CH=N– which was then measured as the concentration of fFN. Through magnetic separation, anti-fFN functionalized magnetic nanoparticles were disseminated in the solution after segregation of unbound anti-fFN.

In order to avoid the interference results from fully or loosely coupled antibodies, oversaturated antibodies (according to the molar ratio between antibodies and magnetic nanoparticles) were used to fully occupy the surface of magnetic nanoparticles during conjugation of the anti-fFN antibodies on the surface of magnetic nanoparticles. After each batch of conjugation, we used the same concentration of analytes (the standard fFN proteins) as quality control materials to make sure the variation of IMR measuring results (the measured concentrations of fFN) is less than 5%, which means every conjugation is stable among each other. The effect of nanoparticle concentration on IMR signals was also investigated. According to the reported paper^21^, it was found that IMR signals increase with increasing saturated magnetic concentrations, achieve a maximum IMR signal, and then decrease. This revealed the fact that there is a definite nanoparticle concentration to attain a maximum IMR signal.

**Figure 1:**
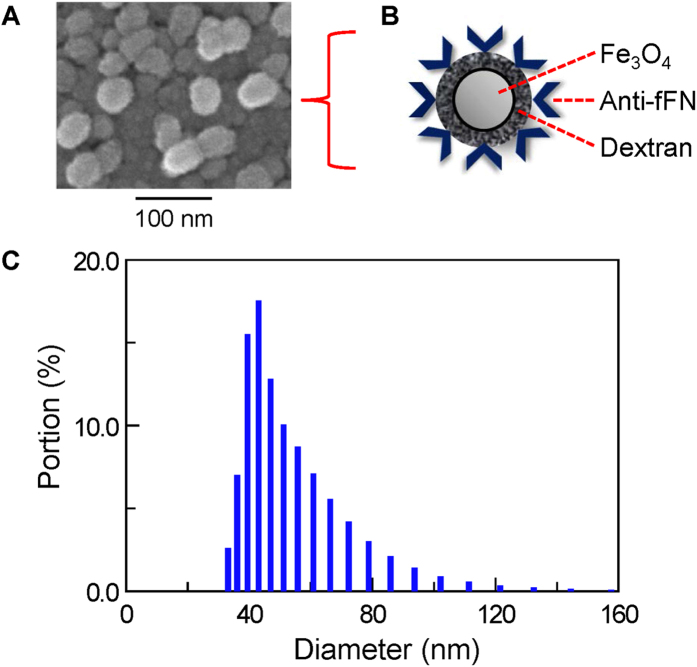
Characterization of anti-fFN magnetic reagent. (**A**) Representative a SEM image (100,000X) of anti-fFN magnetic reagent. The bar below the image indicates 100 nm. (**B**) Illustration of a magnetic Fe_3_O_4_ nanoparticle coated with dextran and anti-fFN antibodies. (**C**) Size and distribution of anti-fFN magnetic reagent determined by DLS.

The size distribution of the anti-fFN functionalized magnetic nanoparticles was analysed using DLS, which showed that their mean (standard deviation) hydrodynamic diameter was 50.50 nm (12.84 nm). Figure 1A shows the image by scanning electron microscope (SEM; JSM-6700F, JEOL, Tokyo, Japan), which was acquired under dehydrated condition. Figure 1B shows the illustration of an anti-fFN coated magnetic nanoparticle. The nanoparticle size in solution was measured by DLS. Figure 1C shows that 68.2% of the magnetic nanoparticles had diameters measuring from 37.66 nm to 63.34 nm. The magnetic concentration of the reagent was 8 mg-Fe/mL, and it was stored at 4 °C before use.

### IMR assay for fFN

**Figure 2:**
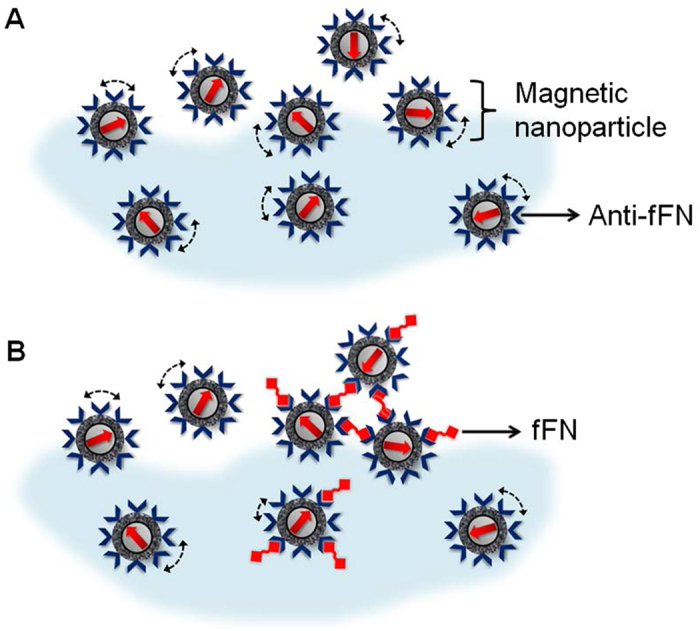
Illustration of the association between fFN biomarkers and magnetic nanoparticles coated with anti-fFN antibodies in an IMR assay. (**A**) All magnetic nanoparticles rotated freely and accordantly with the applied external alternating current magnetic fields before binding with fFN. (**B**) After mixing with assayed samples, some magnetic nanoparticles became larger or clustered because of binding with targeted fFN. Fewer free rotated magnetic nanoparticles donated lower magnetic χ_ac_ signals.

Magnetic nanoparticles coated with anti-fFN were well dispersed in PBS buffer without precipitation or sedimentation. The magnetic reagent showed superparamagnetic characterization when existing in PBS buffer^18^. The magnetic reagent represented the magnetic feature or signal only under an environment applied with a magnetic field. Under external alternating current (AC) magnetic fields, the free magnetic nanoparticles began to rotate (Fig. 2A). The magnetic reagent then demonstrated a magnetic property, called mixed-frequency AC magnetic susceptibility (χ_ac_). The χ_ac_ was expressed as χ_ac,o_ before the magnetic nanoparticles had bound to the targeted fFN. After the reaction of 40 μl of magnetic reagent mixed with 60 μl of sample solution, the magnetic nanoparticles bound to the targeted fFN, resulting in the immune complexes becoming larger and clustered (Fig. 2B). The larger/clustered magnetic nanoparticles rotated slowly leading to attenuation of χ_ac_, defined as χ_ac,ϕ_. The final IMR signal was obtained by calculating the attenuation percentage of the χ_ac_ signal and calculated using the equation:


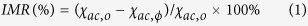


An IMR reader (XacPro-E; MagQu, New Taipei City, Taiwan) was used to detect the real-time IMR χ_ac_ signal at 25 °C. IMR signals were measured in duplicate for each sample. Full-length fFN protein (ab168885; Abcam, Cambridge, UK) was used to establish the calibration curve between the IMR signals and fFN concentration. The concentration of fFN solution ranged from 0.001 ng/mL to 1000 ng/mL.

### ELISA for fFN

A commercial ELISA kit (ab108847; Abcam, Cambridge, UK) was used to assay fFN. The optical density at a wavelength of 450 nm (OD450) was detected using an ELISA reader (Synergy HT; Bio-TEK, Winooski, USA) following the manufacturer’s protocol.

### Statistical analysis

Statistical analyses were performed using SPSS version 24.0 (SPSS, Chicago, IL, USA). Receiver operating characteristic (ROC) curve analysis was used to illustrate the optimum cut-off point to predict a preterm birth for the group with higher IMR or ELISA signals. The optimum cut-off point was defined as the closest point on the ROC curve to the point (0, 1), i.e. sensitivity of 100% and false positive rate of zero.

## Results

### Time dependent χ_ac_ signal

**Figure 3:**
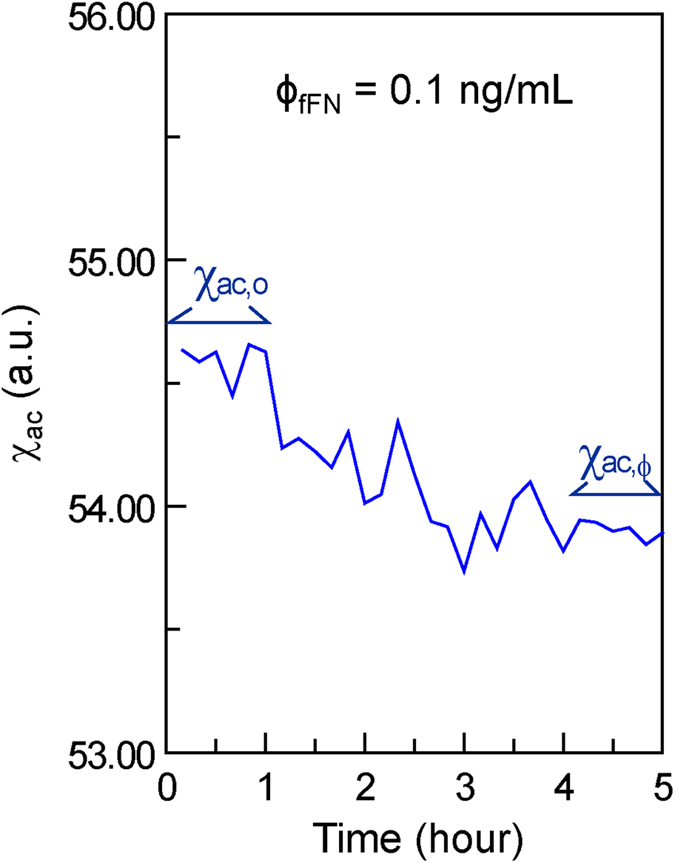
Real-time χ_ac_ signal recorded with an IMR assay on 0.1 ng/mL of standard fFN protein.

After immobilization by binding fFN proteins, the bioactivity of the magnetic nanoparticles was examined using an IMR assay. The time dependent AC magnetic susceptibility χ_ac_ of the magnetic reagent mixed with 0.1 ng/mL fFN solution is shown in Fig. 3. The data from 0 to 1 hour indicated the initial magnetic response χ_ac,o_ of the mixture before incubation. One hour later, χ_ac_ started to reduce, and finally reached equilibrium after 4 hours. The IMR signal as calculated with eq. (1) was 1.29%. The reduction in χ_ac_ of the magnetic reagent suggested an association between the anti-fFN functionalized magnetic nanoparticles and fFN proteins.

### fFN concentration-dependent IMR signal

**Figure 4:**
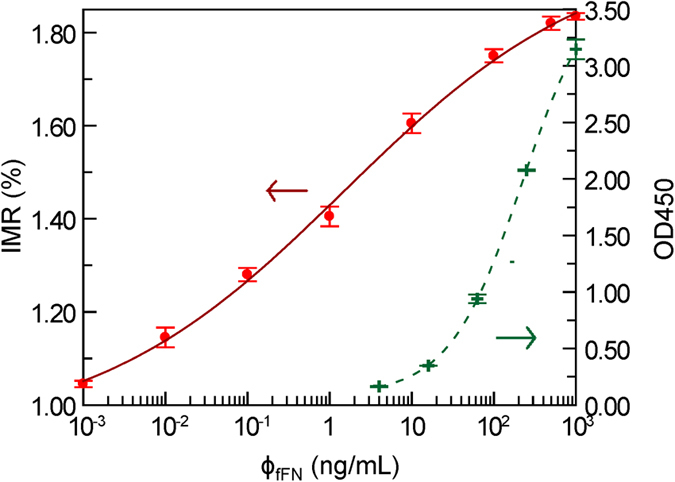
The standard curve of IMR assay (IMR (%); red dots) and ELISA (OD450; green cross symbols) of fFN measurements. The fitting curves using eq. (2) with IMR (%) and OD450 shown as solid red lines and dashed green lines, respectively.

We next investigated the IMR signal as a function of fFN concentration ϕ_fFN_. The fFN concentrations ranged from 0.001 ng/mL to 1000 ng/mL, and IMR signals were measured in duplicate to obtain the mean and standard deviation of each fFN concentration. The measured fFN concentration-dependent IMR signals are shown as red dots in Fig. 4, with the error bars denoting the standard deviation. As the fFN concentrations increased, the IMR signals also increased (from 1.05% ± 0.007) to (1.84% ± 0.007). The data fitted the following four parameter logistic equation^13^:


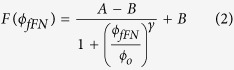


where F(ϕ_fFN_) is the dependent IMR signal (%), A is the minimal signal, B is the maximal signal, ϕ_fFN_ is the fFN concentration, ϕ_o_ is the concentration of the inflection point, and r is the slope at the inflection point of the calibration curve. By fitting the measured fFN concentration-dependent IMR signals in Fig. 4 to eq. (2), the values of A, B, ϕ_o_, and γ were 0.93, 1.99, 1.52, and 0.28, respectively. The curve is shown as the solid red line in Fig. 4. The coefficient of determination R^2^ was 0.998.

### Low detection limit of the IMR assay for fFN

The parameter A in eq. (2) denotes the noise level for the IMR signal, i.e. the IMR signal at ϕ_fFN_ equal to zero. The low detection limit was defined as the lowest concentration recorded with an IMR signal after deducting the noise level using triple standard deviation (3-σ criterion). The standard deviation of the IMR signal at a low concentration of fFN (i.e. 0.01 ng/mL) was 0.021%. Thus, the low detection limit of the IMR signal was (0.93% + 3 × 0.021) = 0.993%. Using eq. (2), the theoretical low detection limit to assay fFN using IMR was around 0.0001 ng/mL.

### Linearity and dynamic range of the IMR assay for fFN

**Figure 5:**
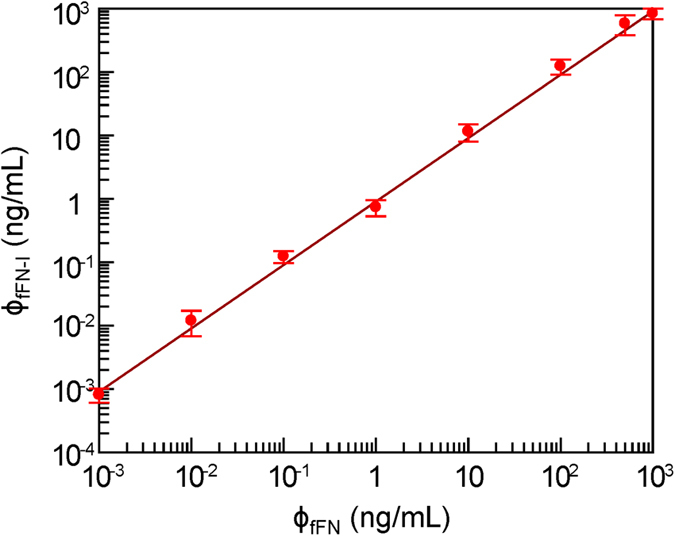
The correlation between spiked fFN (ϕ_fFN_) and measured fFN (ϕ_fFN-I_) by IMR assay.

The IMR signals of various fFN concentrations were converted to fFN concentrations using eq. (2) and denoted as ϕ_fFN-I_. The converted fFN concentration ϕ_fFN-I_ versus the spike of fFN concentration ϕ_fFN_ is shown as dots in Fig. 5. ϕ_fFN-I_ was found to be proportional to ϕ_fFN_ in ϕ_fFN-I_ = 0.904 × ϕ_fFN_. The coefficient of determination R^2^ was 0.971. According to the FDA 510 k regulations, the requirement to determine the range of the linearity in terms of fFN concentration is that the slope exists between 0.9 and 1.1. Figure 5 shows the linearity of assaying fFN using IMR. With the low detection limit of 0.0001 ng/mL, the dynamic range of assaying fFN by IMR ranged from 0.0001 ng/mL to 1000 ng/mL.

### Clinical measurements for the IMR assay of fFN

**Figure 6:**
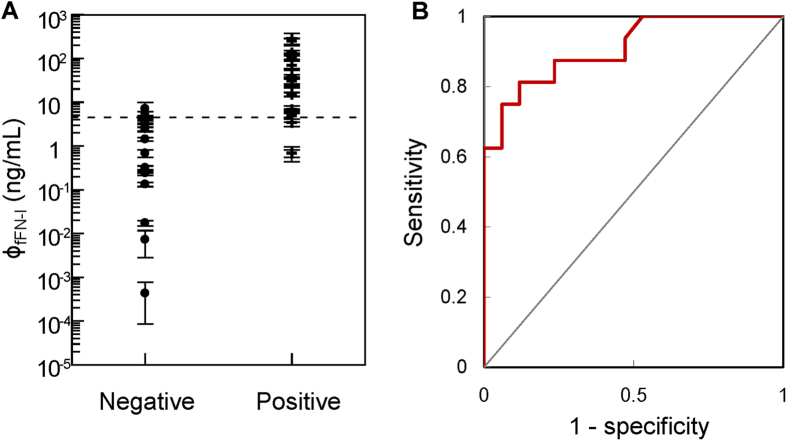
(**A**) Detected fFN concentrations in cervicovaginal secretions using the IMR assay for all subjects, divided into the negative and positive groups. (**B**) ROC curve to determine the cut-off value of fFN concentration to differentiate positive from negative patients. The calculated cut-off value of 5.93 ng/mL is shown as the dashed line in (**A**).

Thirty-three cervicovaginal samples were collected, and the concentrations of fFN were determined by IMR assay. Seventeen samples were categorized into the negative group (gestational age from 37 to 41 weeks, i.e. term birth), and the other 16 samples were categorized into the positive group (gestational age from 24 to 36 weeks, i.e. preterm birth). A positive correlation was found between fFN concentration-dependent IMR signals and preterm birth (Fig. 6A). The fFN concentrations ranged from 0.0001 ng/mL to 10 ng/mL in the negative group, and 0.7 ng/mL to 300 ng/mL in the positive group. Using ROC curve analysis, the threshold of fFN concentration for a laboratory diagnosis of preterm birth was 5.93 ng/mL (Fig. 6B). The area under ROC curve for IMR assay was calculated to be 0.910. The corresponding sensitivity and specificity were 0.833 and 0.944, respectively.

### fFN concentration-dependent OD450 level and low detection limit of OD450

The fFN concentration ϕ_fFN_ dependent OD450 levels are shown as green cross symbols in Fig. 4. The experimental data were fitted to eq. (2), and the values of parameters A, B, ϕ_o_, and γ were 0.088, 3.84, 0.98, and 221.37, respectively. The curve is shown as the dashed green line in Fig. 4. The coefficient of determination R^2^ was 0.999. According to the results shown in Fig. 4, the low detection limit in terms of OD450 was 0.103 according to the 3-σ criterion. Using eq. (2) for ELISA, the low detection limit of fFN concentration was 0.789 ng/mL.

### Linearity and dynamic range of ELISA for fFN

**Figure 7:**
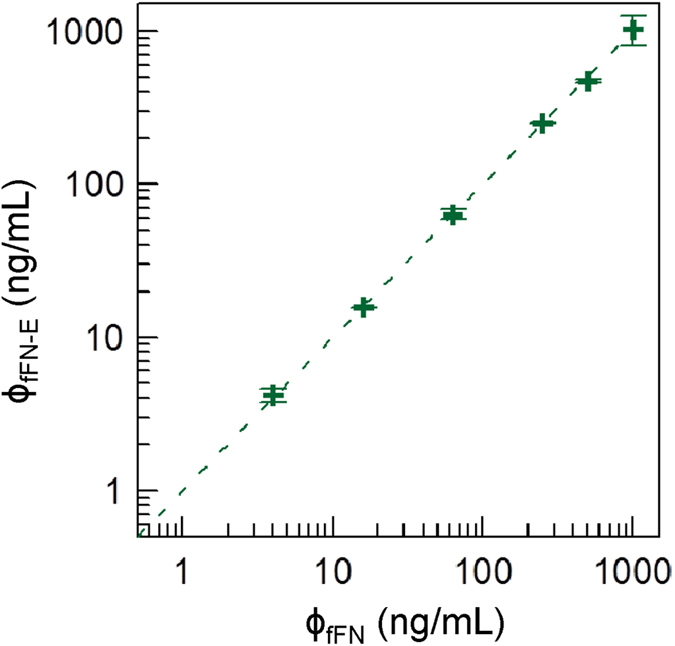
The correlation between spiked fFN (ϕ_fFN_) and measured fFN (ϕ_fFN-E_) by ELISA.

Using eq. (2) for ELISA, the detected OD450 values for various fFN concentrations (from 0 to 1000 ng/mL) were converted to fFN concentrations and denoted as ϕ_fFN-E_. The relationship between the detected fFN concentrations ϕ_fFN-E_ using ELISA and the spike of fFN concentration ϕ_fFN_ is shown in Fig. 7. The coefficient of determination R^2^ was 0.999. ϕ_fFN-E_ was found to be proportional to ϕ_fFN_ with a proportional constant of 1.00, which is within the acceptable range of 0.9 to 1.1 as regulated by the FDA. Hence, the linearity for assaying fFN by ELISA was up to 1000 ng/mL. With the low detection limit of 0.789 ng/mL, the dynamic range of assaying fFN using ELISA ranged from 0.789 ng/mL to 1000 ng/mL.

### Clinical measurements of ELISA for fFN

**Figure 8:**
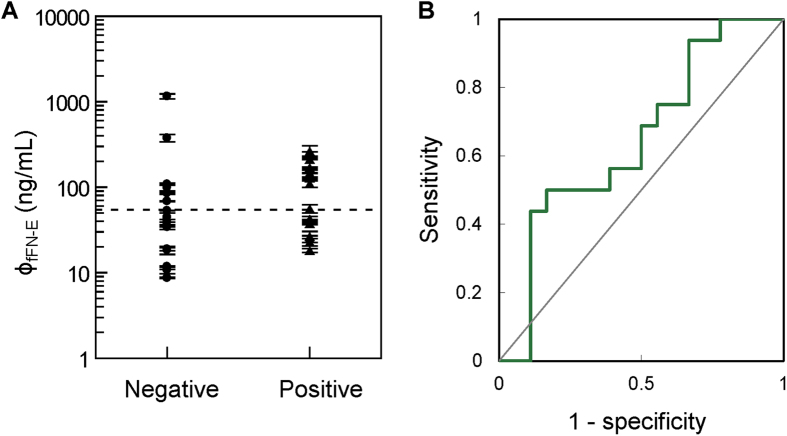
(**A**) Detected fFN concentrations in cervicovaginal secretions using ELISA for all subjects, divided into the negative and positive groups. (**B**) ROC curve to determine the cut-off value of fFN concentration to differentiate positive from negative patients. The calculated cut-off value 54.49 ng/mL is shown as the dashed line in (**A**).

The 33 samples of cervicovaginal secretions collected for IMR assay of fFN were also tested using ELISA. The detected fFN concentrations ϕ_fFN-E_ by ELISA are shown in Fig. 8A, and ranged from 8 ng/mL to 1150 ng/mL in the negative group, compared to 20 ng/mL to 250 ng/mL in the positive group. It seems that there was no clear threshold between the two groups. Through ROC curve analysis (Fig. 8B), the threshold for diagnosing preterm birth by assaying fFN in cervicovaginal secretions using ELISA was 54.49 ng/mL, which is compatible with the cut-off value (50 ng/mL) of commercial kits^11^. The area under ROC curve for ELISA was calculated to be 0.658. The corresponding sensitivity and specificity were 0.583 and 0.611, respectively.

## Discussion

In the IMR assay, magnetic nanoparticles coated with anti-fFN are well dispersed in PBS buffer due to the homogeneous nano-size of nanoparticles. Because of the thermal motion of nanoparticles, the directions of magnetization of nanoparticles are isotropic, which results in zero magnetization under zero applied magnetic fields. Once an external magnetic field is applied to the reagent, magnetization of each nanoparticle tends to be aligned along the applied magnetic field. Consequently, a non-zero magnetization is induced with the reagent. The non-zero magnetization vanishes when the applied magnetic field is removed because magnetization of nanoparticles becomes isotropic. Such magnetic feature is so-called superparamagnetism^18^. It is worthy that the magnetization is induced with the reagent under external magnetic fields via the magnetic driven on nanoparticles by magnetic fields. Dextran, a long chain hydrophilic glucose polymer in which the linkages are predominantly of the α(1, 6) type, is a remarkable candidate for coating on Fe_3_O_4_ nanoparticles to prevent the formation of large aggregates. There are many studies using dextran-coated Fe_3_O_4_ nanoparticles in biomedical applications^14^,^15^,^16^,^17^. Previous researches investigated the magnetic properties of the uncoated and coated dextran Fe_3_O_4_ nanoparticles, and vibrating sample magnetometer (VSM) analysis showed the superparamagnetism of dextran-coated Fe_3_O_4_ nanoparticles^18^,^22^.

Comparisons of the IMR assay and ELISA to determine fFN concentrations are listed in Table 1. Several notable features were found with IMR assay. Firstly, although the upper limits (~1000 ng/mL) of the dynamic range for assaying fFN were similar between the two methods, a much lower fFN concentration could be detected by IMR assay. This implies IMR shows a higher sensitivity in assaying fFN. According to previous papers, the ultra-sensitivity of assaying fFN using IMR is mainly attributed from two factors^15^,^23^. One is the homogeneous assay by utilizing well suspended antibody-functionalized magnetic nanoparticles, the other is the effective suppression in interferences resulted from non-specific molecules and sample colours. The details are explained as follows.

The IMR assay is achieved with homogeneous suspension of anti-fFN functionalized magnetic nanoparticles in the reagent, so that fFN molecule at anywhere in a liquid sample can bind to magnetic nanoparticles to initiate the formation of immune complex magnetic-nanoparticle-anti-fFN-fFN. As to ELISA, only the fFN molecules colliding with the capture antibodies at the bottom of a test well can be sensed in ELISA and not the other fFN molecules suspended far from the bottom of a test well. On the other hand, by utilizing nanoparticles in IMR assay, the total area of immuno-reaction catching fFN molecules is extremely large^15^. It has been estimated that the total surface area of antibody-functionalized magnetic nanoparticles in 1-mL reagent is around 1000 cm^2^. However, the reacting area of a well in a 96-well ELISA plate is 0.45 cm^2^. Thus, the effectively reacting area of a vial for IMR assay is 180 times as that of 96-well ELISA.

In addition to larger area binding with target molecules, the signals due to the binding of capture antibodies and target molecules are more pronounced in IMR assay. The size of the magnetic nanoparticles used in IMR assay was found to be similar to the fFN molecule. When binding between the magnetic nanoparticles and fFN molecules occurred, the effective diameter of the immune complex (magnetic-nanoparticle-anti-fFN-fFN) was substantially larger than the unbound magnetic nanoparticles. The significant expansion in size of the magnetic nanoparticles due to the formation of the immune complex definitely contributed to the appreciable IMR signal. Therefore, the IMR assay shows ultra-high sensitivity in detecting molecules of interest.

The other cause of high sensitivity with IMR assay is the suppression of non-specific binding, which usually results in high background level or false signals. The bound molecules on a magnetic nanoparticle are acted with a centrifugal force because the magnetic nanoparticle is oscillating during the measurement of IMR signals. The centrifugal force can be enhanced by increasing the frequency of particle oscillation^15^. By suitably adjusting the oscillation frequency (~20 kHz in this work), the centrifugal force is stronger than the binding force between antibody and non-specific molecules, whereas is weaker than that between antibodies and target molecules. Hence, the non-specific binding of molecules onto anti-fFN functionalized magnetic nanoparticles is broken out. Background level and false signals due to non-specific binding of magnetic nanoparticles can be significantly eliminated.

In ELISA, optical signals such as optical absorption, transmittance, or fluorescence are detected. Such optical signals shall be easily affected by sample colours generated with haemoglobin, billrirubin, or lipid in a liquid sample. But for IMR assay, magnetic signals instead of optical signals are probed. Magnetic signal is transparent to any kind of colours. Apparently, the IMR assay shall show a much lower background level for signals.

Secondly, the threshold for a laboratory diagnosis of preterm birth by measuring fFN in cervicovaginal secretions using IMR assay (5.93 ng/mL) was much lower than the threshold of ELISA (54.49 ng/mL). Once the highly sensitive and specific IMR assay was used to quantify low concentrations of fFN in the cervicovaginal secretions, the actual low fFN concentrations were revealed. While the poor detection limit of ELISA may cause over quantification of the molecules of interest, resulting in limitations in clinical application.

Thirdly, our results also showed that the accuracy of the diagnosis of preterm birth via assaying fFN in cervicovaginal secretions was much improved when using the IMR assay instead of ELISA. The area under ROC curve in Fig. 6B for IMR assay was calculated to be 0.910, while 0.658 was obtained for the area under ROC curve in Fig. 8B for ELISA.

In this study, the IMR assay demonstrated a good low detection limit of fFN concentration in cervicovaginal secretions, with high sensitivity and specificity. The IMR assay is therefore a potentially promising alternative method to ELISA to accurately predict the risk of preterm birth. A positive fFN result in the IMR assay can alert clinicians to treat high-risk patients as quickly as possible, such as the administration of tocolytic agents or corticosteroids to mature the foetal lungs, and early referral to a tertiary medical centre with neonatal intensive care units which offer a higher level of care for preterm new-borns. On the other hand, a negative fFN result may reduce the anxiety of the parents and avoid unnecessary medical interventions.

## Additional Information

**How to cite this article:** Wong, C.-H. *et al*. Bio-functionalized magnetic nanoparticles for the immunoassay of foetal fibronectin: a feasibility study for the prediction of preterm birth. *Sci. Rep.* **7**, 42461; doi: 10.1038/srep42461 (2017).

**Publisher's note:** Springer Nature remains neutral with regard to jurisdictional claims in published maps and institutional affiliations.

## Figures and Tables

**Table 1:** Specifications of assaying foetal fibronectin in cervicovaginal secretions using IMR assay and ELISA.

Method	LDL (ng/mL)	DR (ng/mL)	Threshold (ng/mL)	Sensitivity	Specificity
IMR	0.0001	0.0001–1000	5.93	0.833	0.944
ELISA	0.789	0.789–1000	54.49	0.583	0.611

LDL: low detection limit; DR: dynamic range.
